# Infant and Young Child Feeding practices up to 23 months in Namuno District, Cabo Delgado, Mozambique

**DOI:** 10.1186/s13052-023-01517-5

**Published:** 2023-09-01

**Authors:** Kodak Raúl Marroda, Cristiana Berti, Adriano La Vecchia, Carlo Agostoni, Bruno Nazim Baroni, Silvia Bettocchi, Mattia Baglioni

**Affiliations:** 1 AVSI Foundation Mozambique, Pemba, Mozambique; 2 Fondazione IRCCS Ca’ Granda Ospedale Maggiore Policlinico, Milan, Italy; 3 Department of Clinical Sciences and Community Health, University of Milan, Milan, Italy; 4 Fondazione De Marchi, Milan, Italy

**Keywords:** Breastfeeding, Complementary feeding, Dietary diversity, Livelihood activities, Delivery health facility

## Abstract

**Background:**

Inadequate breastfeeding and complementary feeding practices are key determinants of child malnutrition and impact one-third of the under-five mortality rate worldwide. The district of Namuno in Cabo Delgado (Mozambique) has been long registering a high prevalence of acute malnutrition. To date, no data exists about Infant and Young Child Feeding (IYCF) practices in the area. The current pilot study explores the feeding practices among children aged 0–23 months in Namuno and sets out its main drivers.

**Methods:**

This cross-sectional study was realized between August and September 2019 and drew on questionnaires for mothers/caregivers of children aged 0 to 23 months. We computed IYCF indicators and correlated them with mothers’/caregivers' school attendance, delivery setting, and distance between home and the place where livelihood activities took place (workplace), using univariate and multivariate (age-adjusted) logistic regression analysis.

**Results:**

The survey was data derived from a sample of 632 mothers/caregivers. 62% of 0–23-month-old children received colostrum whilst only 31% of 0–5 months babies were on exclusive breastfeeding (EBF). Among 6–23 months old children, 17% consumed foods and beverages from at least five out of eight defined food groups, 31% received a minimum frequency of meals, and 23% had a minimum acceptable diet. Data revealed a positive correlation between early initiation of breastfeeding and delivery in a maternity ward (OR 6.9, CI 3.2–16.1, *p*-value < 0.001). No difference in the IYCF indicators between female and male babies was detected.

**Conclusions:**

In the Namuno district, IYCF practices did not fulfill WHO/UNICEF’s indicators and recommendations. This suggests that efforts should be focused on EBF-enabling interventions to improve children’s dietary consumption patterns.

## Background

Breastfeeding and complementary feeding practices are key determinants of malnutrition that in turn affect one-third of the under-five mortality rate [1]. Feeding practices influence the health, growth, development, and nutritional status of children aged 0–23 months. The World Health Organization (WHO) and the United Nations International Children’s Emergency Fund (UNICEF) defined 17 indicators on Infant and Young Child Feeding (IYCF) to shape policy-making and monitor progress on feeding practices at the national and the global level [2]. Worldwide, only 42% of children aged 0–6 months are breastfed and 29% of children aged 6–23 months eat foods from the minimum number of food groups [3, 4]. The IYCF indicators for developing countries show the most worrying trends for children under 24 months of age and put in evidence the growing issue of social inequities at the origin of food insecurity [5–8].

In Mozambique, the indicators concerning exclusive breastfeeding (EBF) and complementary feeding (CF) give a reason for concern. The latest available national data dated back to 2011 [9] pointed out that only 43% of children aged 0–5 months were exclusively breastfed, 28% of children aged 6–23 months received foods from four or more food groups, 44% had the Minimum Meal Frequency (MMF), and 15% had the minimum frequency of foods including at least four or more food groups (the so-called Minimum Acceptable Diet [MAD]). Recent reports and analyses carried out by the specialized institutions of the Government of Mozambique and international partners highlighted that Namuno is one of the districts in the country most affected by acute malnutrition and food insecurity. In 2019, 2% of 0–59-month-old children were acutely malnourished by weight-for-height and 7% by mid-upper arm circumference [10].

To date, data about IYCF practices in this area do not exist. Hence, we carried out an explorative study aimed at gathering overall preliminary information on the feeding practices among children aged 0–23 months in the Namuno district. Furthermore, the study examines some of the main causal factors of the suboptimal state of the IYCF indicators by focusing on three objectives:

1. To assess 8 IYCF indicators out of the total 17 introduced by WHO and UNICEF in 2021, notably Early initiation of breastfeeding, EBF under 6 months, Continued breastfeeding at 1 year, Continued breastfeeding at 12–23 months, Introduction of solid or semi-solid or soft foods, Minimum dietary diversity 6–23 months (MDD), MMF 6–23 months, and MAD 6–23 months;
2. To investigate the relationship between IYCF practices and the mothers/caregivers’ level of education; the place of delivery (home or maternity ward); the distance of mothers’/caregivers’ home from the place where their livelihood activities take place (workplace); and the sex of the child. The hypothesis was that IYCF practices improved as the mothers’/caregivers’ level of education increased, the mothers delivered at the hospital, and the mothers/caregivers performed livelihood activities close to their homes.
3. To shed light on myths and taboos affecting infant feeding practices.

Our findings may represent a starting point to inform decisions on the interventions needed to tackle malnutrition rates among children aged 0–23 months in target areas of the province of Cabo Delgado (Mozambique).

## Material and methods

### Setting and study population

This pilot cross-sectional study is based on survey data gathered between August and September 2019 in Namuno, a rural district located in the southern part of Cabo Delgado province that covers an area of approximately 6,000 km^2^. According to the last census carried out in 2017, the population of the district amount to 245,248 individuals and 62,397 households. The Catholic faith is the most predicted one (61%) followed by the Muslim one (34%) and others (5%). The ethnic composition in the Cabo Delgado province (2,267,715) comprises the following main groups: Emakhuwa (66.8%), Shimakonde (21.8%), Kinwani (6.1%). Available data show that the main ethnic group in Namuno is Emakhuwa (around 50%) [11, 12]. The survey sample was selected from eight, out of the hundred-fifty, villages of Namuno (Nicuita, Napila B, Mahossine, Pulupo, Milipone, Nanrapa, Chopa, and Nicane). A non-probability convenience sampling was adopted due to the limited access to local demographic data as well as the time and funding available to carry out the data collection [13]. This study was carried out within the scope of a 2-year development project aimed at improving nutrition-sensitive interventions focused on agriculture and financed by the Italian Agency of Cooperation for Development (*Agenzia Italiana per la Cooperazione allo Sviluppo, AICS*) and implemented by the AVSI Foundation.

Eligible participants were mothers/caregivers of children aged 0 to 23 months. For each family, only the oldest child between 0 to 23 months of age was selected as this was considered to be able to display overall information concerning infant feeding adopted within the household.

The Health Department of the Namuno District collaborated actively with AVSI staff for the realization and implementation of all the nutrition sector-related activities in this study from the outset of the project, including study design development and data collection. Monthly, technical meetings were held to coordinate the implementation of project activities and to inform local health authorities about the results.

### Data collection

Mothers/caregivers were approached at their homes through the mediation of local leaders that invite them to participate in the research. Data collection occurred only thereafter the informed consent was obtained from parents/caregivers. The participants were surveyed through a structured questionnaire based on the National Breastfeeding Survey of Brazil’s "Research Questionnaire on Feeding Practices of Children aged 0–12 months" [14] and created through the tablet-installed Kobo Toolbox application which facilitated data collection. The questionnaire was adapted to the local setting to assess feeding practices for children aged 12–23 months. The assessment of infant feeding practices was performed through a 24-h recall and included specific questions on the introduction of liquid consumption such as water, tea, coffee, juice, juice powder, coconut water, and soft drinks [2, 15]. Socio-demographic data on children, mothers/caregivers, and at the community level were also collected to cast the light on the mother/caregiver’s level of education, the distance between home and place where the mother/caregiver practiced her livelihood activities, information about mothers’ delivery such as place of delivery (home or maternity ward), type of delivery (cesarean section or vaginal), and type of child healthcare (hospital or traditional doctor). Ten enumerators with a health profile were trained for four days to get familiar with the questionnaire uploaded on the tablet.

Myths, traditional beliefs, and taboos influencing feeding practices were explored through focus group discussions based on a semi-structured interview including both “yes–no” and open-ended questions. For each village, two gender-balanced focus groups were set up, with a maximum of 20 participants per group.

### Data analysis

The descriptive statistical analysis was performed by computing frequencies, median, and prevalence, as recommended by WHO [2]. The adherence to minimum standards for feeding practices as established through WHO’s and UNICEF’s definitions [2, 15] (Table 1) was based on the calculation of eight IYCF indicators, four related to BF and four to CF.

**Table 1:** IYCF indicators’ category and definition according to WHO and UNICEF 2021 [2, 13] used in this study

Indicator category	Indicator	Definition
***Breastfeeding indicators***	*Early initiation of breastfeeding*	Proportion of children born in the last 24 months who were put to breast within the first one hour
	*Exclusive breastfeeding under 6 months (EBF)*	Proportion of infants 0–5 months of age who are fed exclusively with breast milk
	*Continued breastfeeding at 1 year*	Proportion of children 12–15 months of age who are fed breast milk
	*Continued breastfeeding 12–23 months*^a^	Percentage of children 12–23 months of age who were fed breast milk during the previous day
***Complementary feeding indicators***	*Introduction of solid or semi-solid or soft foods*	Percentage of infants 6–8 months of age who consumed solid, semi-solid, solid or soft foods during the previous day
	*Minimum dietary diversity 6–23 months (MDD)*	Percentage of 6–23 months of age who consumed foods and beverages at least five out of eight defined food groups during the previous day
	*Minimum meal frequency 6–23 months (MMF)*	Percentage of children 6–23 months of age who consumed solid, semi-solid or soft foods (but also including milk feeds for non-breastfed children) at least the minimum number of times during the previous day. The minimum number is defined as:
		- 2 feedings of solid, semi-solid or soft foods for breastfed infants aged 6–8 months;
		- 3 feedings of solid, semi-solid or soft foods for breastfed children aged 9–23 months;
		- 4 feedings of solid, semi-solid or soft foods or milk feeds for non-breastfed children aged 6–23 months whereby at least one must be a solid, semi-solid or soft feed
	*Minimum acceptable diet 6–23 months (MAD)*	Percentage of children 6–23 months of age who consumed a minimum acceptable diet during the previous day defined as:
		- For breastfed children: receiving at least the minimum dietary diversity and minimum meal frequency for their age
		- For non-breastfed children: receiving at least the minimum dietary diversity and minimum meal frequency for their age

^a^The Authors of this paper considered relevant to include this indicator belonging to the previous edition of the document updated in 2021(7) as it helps to gather breastfeeding information regarding the age 12–23 months

To study the correlation between IYCF indicators and mother’/caregiver’s determinants, we divided the total sample into two groups according to their age, 0–5 months and 6–23 months. Univariate and multivariate (age-adjusted) logistic regression analyses were performed using IYCF indicators as dependent variables. The independent variables were the mother/caregiver’s school attendance (yes/no), the distance between home and the place where the mother/caregiver practiced her livelihoods activities (i.e., a distance greater/minor than 1 km from home), place of delivery (in/out maternity ward), and the child sex. The dependent variables were EBF and early initiation of breastfeeding for 0–5 months children and MDD, MMF, and MAD for 6–23 months children. To control the false discovery rate, the p–values obtained were adjusted using the Benjamini–Hochberg method. A *p*-value less than 0.05 was considered statistically significant. Statistical analysis was performed using R software (version 3.6.3 for Windows).

## Results

### Participants

We interviewed 679 mothers/caregivers. We excluded 47 questionnaires due to fundamental data and information misreporting through the e-questionnaires. Therefore, we included 632 women in the analysis.

Table 2 shows the characteristics of the participants. Almost 60% of mothers/caregivers reported attending school until the 5^th^ primary school grade, while 30% did attend any school. Most women (about 72%) practiced their livelihoods (farming activities) far from their residences.

**Table 2:** Characteristics of the children and their mothers/caregivers included in the study

Population group	Characteristic	n (%)
***Mothers/Caregivers***	*Age in years, Median [range]*	26 [13-64]
	≤ 17 years	42 (7)
	≥ 18 years	565 (89)
	n.a	25 (4)
	*School attendance*	
	5^th^ primary school grade	367 (58)
	Between 6^th^-7^th^ primary school grade	64 (10)
	Between 8^th^-12^th^ primary school grade	13 (2)
	Never	188 (30)
	*Maternal livelihood activity*	
	Farmer	628 (99)
	Trader	2 (0.3)
	Homemaker	2 (0.3)
	*Distance of livelihood activities from home*	
	Distant (> 1 km)	453 (72)
	Nearby (< 1 km)	172 (27)
	Lying-in	7 (1)
***Children***	*Sex*	
	Female	330 (52)
	Male	302 (48)
	*Age group*	
	0–5 months	162 (26)
	6–23 months	473 (74)
	*Place of delivery*	
	In the maternity ward	320 (51)
	Out of the maternity ward	312 (49)
	*Type of delivery*	
	Vaginal	632 (99)
	Cesarean section	9 (3)
	*Birth weight*^a^	
	< 2500 g	51 (16)
	≥ 2500 g	269 (84)
	*Service for childcare consultation*	
	Health facilities	602 (95)
	Traditional doctors	15 (2)
	Any service	15 (2)

^a^Data derived only from mothers who delivered in the maternal ward (*n* = 320)

Almost half of the children were delivered outside the maternity ward; 3% were born by cesarean section, and 16% were born with low birth weight. Most childcare health consultations took place at health facilities. In very few cases, mothers/caregivers resorted to traditional medicine, or no consultation was registered. No child attended daycare centers.

### Feeding practices

Table 3 gives the total number and the percentage of children that consumed food items from the eight food groups regardless of the appropriate age for complementary feeding. Diets including protein-rich food groups, such as pulses, flesh foods, and eggs were around 56% (*n* = 358), 17% (*n* = 110), and 1% (*n* = 7) respectively; the consumption of Vitamin-A-rich fruits and vegetables was close to 32% (*n* = 204).

**Table 3:** Food items consumed according to the 24 h recall

Food group	Food items	N° consuming the food items	%
*Breast milk*	Breast milk	605	96
*Grain, roots, tubers, plantains*	Rice, sorghum, yam, millet, plantain, cassava, maize, pasta	444	70
*Pulses*	Beans, peas, lentils, cashew nut, sesame, groundnut, sunflower seeds	358	57
*Dairy products*	Processed milk, milk from animals, infant formula, cheese	16	3
*Flesh foods*	Fish, chicken, pork, sheep, goat, liver and other organ meats, duck, beef, lamb, processed meat	110	17
*Eggs*	Eggs	7	1
*Vitamin-A rich fruits and vegetables*	Pumpkin, squash, carrot, orange-flashed sweet potato, sweet potato, dark green leafy vegetables	204	32
*Other fruits and vegetables*	Banana, orange, papaya, manga, *massala* (*Strychnos Spinosa*), other seasonal fruit	101	16

Table 4 summarizes data on IYCF practices by age group. More than half of children aged 0–23 months received breast milk within the first hours of life (the colostrum) as recommended by WHO. The rate of children under 6 months exclusively breastfed was 31%, while 64% were breastfed within an hour of birth. The very early age at which fluids such as water, powdered juices, or solid, and semi-solid foods were introduced was 3.0 ± 1.6 months (data not reported in the table). Among children aged 12–15 months, all children continued to receive breast milk until 1 year of age. By including the information regarding the age 12–23 months (data not shown in the table), the percentage of breastfed children was around 43% (*n* = 272). Among 6-month-old children, the consumption of other types of milk took place in 9 cases (2%), while 4% (*n* = 26) weaned children older than 6 months received other milk less frequently (data not presented in the table). Solid, semi-solid, or soft foods were given to about 70% of children aged between 6 and 8 months. Regarding the quality of CF, only 17% received the MDD while 30% had the MMF among 6–23 months-old children. When combining MDD and MMF, only 6% consumed food items from at least 5 food groups in acceptable frequency (the so-called MAD).

**Table 4:** Infants adhering to each Infant and Young Child Feeding (IYCF) indicator stratified according to the age category

**IYCF indicators**		**Age-based group**		
***Feeding Practice***	***Age group (months)***	***N tot***	***N° adhering to feeding practice***	***%***^a^
*Early initiation of breastfeeding*	0–23	632	391	62
*Early initiation of breastfeeding*	0–5	162	104	64
*EBF under 6 months*	0–5	162	50	31
*Continued breastfeeding at 1 year*	12–15	109	109	100
*Introduction of solid, semi-solid, or soft foods*	6–8	114	76	67
*Minimum dietary diversity*	6–23	470	81	17
*Minimum meal frequency*	6–23	470	145	31
*Minimum acceptable diet*	6–23	470	26	6
*Continued breastfeeding at 2 years*	20–23	70	51	73

^a^Percentage calculated as the ratio between the number of children within the specific IYCF Indicator age group and the total number of children, independently of the age, adhering to that specific IYCF Indicator

### Association between IYCF practices and the mothers/caregivers’ determinants

**Fig. 1:**
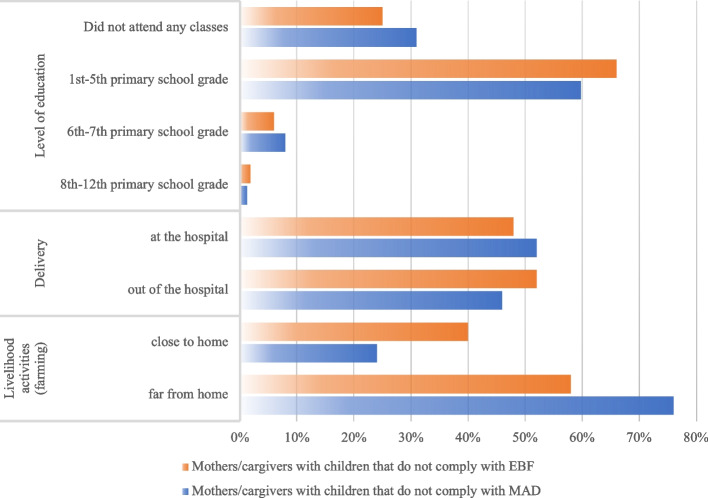
Prevalence of exclusive breastfeeding (EBF) and Minimum acceptable diet (MAD) in relation to mothers’/caregivers’ level of education, delivery place, and distance from home to the place of the mother/caregiver’s livelihood activity (farming). The figure highlights the non-compliance with EBF and MAD by qualitatively cross-checking these two Infant and Young Child Feeding (IYCF) indicators with the three mothers/caregivers-related variables, i.e., level of education, place of delivery, and distance between livelihood activities’ place and home

Figure 1 shows the prevalence of participants adhering to the EBF and MAD according to WHO and UNICEF’s standards with the three variables, i.e. the level of education of mothers/caregivers, the setting of delivery, and the distance from their home to the place where the livelihood activities were practiced.

The exclusively breastfed children aged 0–5 months were significantly younger than not-exclusively breastfed ones (median age 1 vs 3 months respectively, *p*-value < 0.01). The delivery in a maternity ward was positively associated with early initiation of breastfeeding (OR 6.9, CI 3.2–16.1, *p*-value < 0.001). After adjusting the *p*-values, we did not find any correlation between the level of education of mothers/caregivers, sex, the distance between the work-place from home, and EBF.

We did not find any correlation between the IYCF indicators for 6–23 months of children and the mothers’/caregivers’ determinants. Table 5 shows the results of univariate and multivariate logistic regression analyses with adjusted *p*-values.

**Table 5:** Odds ratios and 95% confidence intervals between Infant and Young Child Feeding (IYCF) indicators and mothers’/caregivers’ determinants by age groups. The p-values are adjusted using the Benjamini–Hochberg method

Dependent variable	Independent variable	OR	CI 95%	*p*-value
**Children 0–5 months: univariate analyses**				
EBF	Delivery in a Maternity Ward	0.5	0.2–1	0.3
	Mother attended school	0.9	0.4–2.1	1
	Distant workplace from home (> 1 km)	1.4	0.7–3	0.6
	Male sex	1	0.5–2	1
Early initiation of breastfeeding	Delivery in a Maternity Ward	6.9	3.2–16.1	< 0.001
	Mother attended school	0.7	0.3–1.6	0.4
	Distant workplace from home (> 1 km)	1	0.5–2	1
	Male sex	0.6	0.3–1.3	0.5
**Children 0–5 months: age-adjusted analyses, age in months (mean ± SD) = 2.5 ± 1.7**				
EBF	Delivery in a Maternity Ward	0.4	0.2–0.9	0.09
	Mother attended school	0.6	0.2–1.4	0.3
	Distant workplace from home (> 1 km)	2.2	1–5	0.09
	Male sex	1	0.5–2.1	1
Early initiation of breastfeeding	Delivery in a Maternity Ward	7	3.2–16.3	< 0.001
	Mother attended school	0.7	0.3–1.6	0.4
	Distant workplace from home (> 1 km)	0.9	0.4–2	0.9
	Male sex	0.6	0.3–1.3	0.6
**Children 6–23 months: univariate analyses**				
MDD	Delivery in a Maternity Ward	1.5	0.9–2.5	0.4
	Mother attended school	0.8	0.5–1.4	0.7
	Distant workplace from home (> 1 km)	1	0.6–1.9	0.8
	Male sex	1.3	0.8–2.1	0.4
MMF	Delivery in a Maternity Ward	1	0.6–1.4	0.9
	Mother attended school	1	0.6–1.5	0.9
	Distant workplace from home (> 1 km)	0.9	0.6–1.4	0.9
	Male sex	1.4	0.9–2.1	0.32
MAD	Delivery in a Maternity Ward	1.4	0.6–3.3	0.8
	Mother attended school	1	0.4–2.5	1
	Distant workplace from home (> 1 km)	1.1	0.4–3	1
	Male sex	2.5	1.1–6.2	0.12
**Children 6–23 months: age-adjusted analyses, age in months (mean ± SD) = 13.8 ± 5.0**				
MDD	Delivery in a Maternity Ward	1.5	0.9–2.5	0.4
	Mother attended school	0.8	0.5–1.4	0.7
	Distant workplace from home (> 1 km)	1.1	0.6–1.9	0.8
	Male sex	1.3	0.8–2.2	0.4
MMF	Delivery in a Maternity Ward	0.9	0.6–1.4	0.8
	Mother attended school	1	0.6–1.5	0.8
	Distant workplace from home (> 1 km)	0.9	0.6–1.4	0.8
	Male sex	1.4	0.9–2.1	0.32
MAD	Delivery in a Maternity Ward	1.4	0.6–3.3	0.8
	Mother attended school	1	0.4–2.5	1
	Distant workplace from home (> 1 km)	1.1	0.4–3	1
	Male sex	2.5	1.1–6.2	0.12

*EBF* exclusive breastfeeding, *CI95%* confidence interval 95%, *MAD* minimum acceptable diet, *MDD* minimum diet diversity, *MMF* minimum meal frequency, *OR* odds ratio, *SD* standard deviation

### Tradition regarding feeding practices

Table 6 reports a transcript of some of the statements regarding traditions, beliefs, and taboos applied to food consumption. Information was obtained about prohibitions and restrictions related to the intake of certain single food items in childhood, pregnancy, and lactation. Eggs, bananas, *Moringa*, liver, honey, and sugarcane were considered potentially harmful foods, with eggs and sugarcane thought to exert detrimental effects on the fetus during pregnancy.

**Table 6:** Statements on myth and taboos towards the consumption of specific food items in children under 2 years of age, pregnant and lactating women gathered during the focus group discussions

Type of food	Statement
*Egg*	“When the child eats it causes stomachache and defecates feces the color of yolk and egg white”
	"When a pregnant woman eats it, a hairless baby is born"
*Banana*	"When the child eats it causes general weakness and increases the volume of the abdomen, may have diarrhoea, also the skin becomes pale"
*Moringa leaves*	"When the child eats, they get stomach pains, reddened eyes, abdominal cramps, tonsillitis and bad mood"
*Liver of all kinds of animals*	"When the child eats it, he feels stomach pains and defecates dark stools"
	"When the child eats it, he gets very sick, vomits of yellowish color and dizziness"
*Leftovers from the previous day*	"When the child eats it causes symptoms of malaria and abdominal distension"
*Honey*	"When the child eats it makes the skin yellowish and causes anaemia in the child, it can also cause abdominal pain, vomiting and diarrhoea with yellowish feces"
	"When the lactating woman eats it, the child breastfed becomes mentally retarded and defecates honey-colored feces"
*Sugarcane*	"When the pregnant woman eats it, she moans at the time of delivery. The new-born baby trembles and becomes weak during the first week after delivery. A baby is born with reddish spots and itchy eyes"
	"When the lactating woman eats it makes breast milk that causes weakness and stomachache in the baby. The child defecates white feces"

## Discussion

To our knowledge, this is the first study on the prevalence of EBF and CF indicators in the Namuno District. This work set out to provide information on IYCF practices in the area and to put in evidence the relationship between EBF and MAD indicators with three variables related to mothers’/caregivers' characteristics: education, place of delivery, and distance from home to the place where livelihood activities occur.

### IYCF indicators

Based on the classification criteria of the WHO [16], our results reveal that EBF and complementary feeding were not satisfactory, especially when relating to MDD and MAD.

EBF prevalence in children under 6 months (31%) was compromised by the early introduction of fluids or solid, and semi-solid foods, at non-recommended ages (i.e., 3.0 ± 1.6 months). The low prevalence of EBF is a widespread phenomenon in sub-Saharan Africa and ranges from 24% (Central Africa) to 56% (Southern Africa) [17]. Many factors might explain this trend: mothers’ necessity to dedicate time to livelihood activities during the lactation period, the caregivers' misconceptions that their child is old enough, and that breastfeeding alone is insufficient to meet the infant's nutritional needs [18–20]. Our study results revealed that 62% of children received breast milk within the first hours of life. Although this prevalence does not fulfil the WHO recommendation, it was higher than other findings for Sub-Saharan countries. In some developing settings, mothers reportedly discarded colostrum owing to cultural beliefs because it is considered “dirty” and a potential vector for diseases [21, 22]. As to continued breastfeeding at 1 and 2 years, we found a high prevalence of continued breastfeeding with 100% of children being breastfed up to 12 months and almost 73% up to 23 months. This seems a common pattern among low-and middle-income countries (LMICs) due to the high prevalence of food-insecure families [3, 23–25].

Concerning CF, we found that only one out of twenty children aged 0–23 months received an acceptable diversity of food groups at an appropriate frequency. The same trend was observed in other sub-Saharan countries [26]. Only one-fourth of children received animal protein food sources or fruits, and only one-fifth ate iron-rich foods. As expected among resource-poor populations generally consuming monotonous diets [27], we observed that almost 100% of children eat maize and sorghum porridge as the main staples. Only some of these meals were enriched with groundnut, salt, or sugar. The study population did not mix porridge with foods that are sources of proteins, green leafy vegetables, seasonal fruits, and tubers that contain vitamin A. Survey data revealed that the complementary feeding was poor in proteins and micronutrients. Children’s diet was not adequately diversified most likely due to constraints of economic access to markets and a limited set of crops (on average five) cultivated by families in rural areas [28, 29]. Eggs, bananas, honey, and other foods were less offered to children due to myths and taboos, as learned during the focus group discussions. Similarly, Picolo and colleagues [30] recorded mothers’/caregivers’ negative opinions about egg consumption during breastfeeding albeit with different motivations (for instance, inhibition of breast milk production). Anthropological studies in Ethiopia and Tanzania highlighted that IYCF practices reflected cultural norms transmitted within social networks and could not adhere to recommended public health standards [31, 32].

We reported higher adherence to MAD in male than in female children, even if this finding resulted not significant after adjusting the *p*-values. Some previous studies highlighed that male children were more likely to achieve MAD than female children [33–35]. Such a difference might be imputable to socioeconomic or cultural aspects which lead to gender preference and discrimination during the feeding of the child that translates into prioritizing male over female babies. To overcome this trend, more efforts should be made to promote gender equality regarding feeding as a matter of urgency.

### IYCF and mothers’/caregivers’ level of education

Several studies demonstrated that mothers’/caregivers’ access to just primary school had a better impact on adherence to recommended IYCF practices [6, 23, 36]. In contrast, we did not find any correlation between the mother/caregiver school attendance and the IYCF indicators. In our population sample, only 2% of the total mothers/caregivers attended from the 8^th^ to the 12^th^ grade, and more than half of them did not attend classes beyond the 5^th^ primary school grade. Therefore, we did not compare adherence to EBF and MAD across different levels of education. This made it difficult to reach a sufficiently consistent conclusion about the role of education in compliance with UNICEF and WHO’s IYCF standards.

### IYCF and place of delivery

Women should receive counseling about breastfeeding during at least the first 6 months of their children’s life (notably for the first two/three days), including antenatal care [37]. This may have a positive impact, especially on EBF and breastfeeding within the first hour of life. For instance, it was reported that mothers whose delivery was attended by a health professional had 4.75 times higher odds of early initiation of breastfeeding as compared to those who were not [21]. Overall, several investigations point out the importance of delivering at the maternity ward of the health centers and carrying out both antenatal and postnatal care so that mothers can adequately feed their children [38–42]. Yet, data from six African countries, including Mozambique [43], showed that only 43% of the health workers assisted mothers to initiate breastfeeding within the first hours of life. For Mozambique, this indicator was around 20%. Another study in the city of Nampula [18] highlighted the main shortcomings of the health system, its negative impact on breastfeeding practices, and the measures to be taken to promote behavioral change. We found a positive correlation between delivery in maternity wards and early initiation of breastfeeding. However, we did not find a correlation between the delivery in maternity wards and the EBF for children younger than 6 months. This data supports the idea that either training sessions or refreshments for healthcare professionals are needed to possibly transmit the EBF-related key messages and knowledge to the communities.

### IYCF and mothers’/caregivers’ livelihood activities (farming)

Several studies investigated the nexus between feeding practices and either mothers’ employment or general family livelihood activities. Research conducted in Ethiopia and Somaliland [38, 40] pointed to better child feeding when related to increasing household income. When socio-economic determinants of dietary adequacy were analyzed in 80 LMICs, breast milk was the only type of food with a pro-poor distribution. Conversely, animal-source foods showed the most pronounced pro-rich inequality with dietary diversity improving with absolute annual household incomes exceeding ∼US$20,000 [44].

Despite the evidence on the association between mothers’ working hours on breastfeeding [45], a few sources were found regarding the relationship between the distance from mothers/caregivers’ homes and workplaces on the one hand, and infant feeding in extremely poor rural contexts on the other [46, 47]. Considering that our survey took place in a rural setting where agriculture is the main subsistence activity, greater relevance was attributed to the variable "distance from home" to the main livelihood activity, namely small-scale farming. We observed a correlation between distant workplaces (> 1 km) and EBF. In the Namuno district, as in other rural contexts, a distant workplace could be a marker of economic status, i.e. the lack of a family and financially supportive environment. This condition could force the mother to take the child to the place where livelihood activities are carried out, therefore preventing the mother–child separation during work time and favoring EBF.

### Limitations

One of the major limitations of the present study stems from the methodology adopted for data collection which prevents the authors from inferencing the study results to the whole district of Namuno. The survey was carried out only in the eight villages of Namuno where the project implemented by AVSI took place.

Although the evaluation of feeding practices based on the 24-h dietary recall is the most widely used method, it may lead either to overestimation or underestimation of some indicators (especially overestimation of EBF). This is because children who received other liquids or food irregularly may have not received them the day before the interview [15]. Furthermore, the variation of food availability and diversity during lean seasons [10] may lead to differences in dietary consumption patterns throughout the year. This variation may also have an intergenerational impact on mothers’ offspring’s growth as demonstrated by Rickard and colleagues [48]. Finally, the estimates that are based on a limited number of cases (for example, only 91 children between 6 and 8 months), are less accurate than others that refer to the overall sample studied (for example, the indicator "early initiation of breastfeeding" is estimated based on 632 children).

## Conclusions

Our explorative survey found that compliance with IYCF indicators in the Namuno district was not satisfactory. Therefore, actions need to be taken to improve the quality of infant feeding practices through the introduction of food items at the appropriate age also considering adequate diversity and frequency. The delivery at the maternity ward did not improve adherence to IYCF indicators, except for the early initiation of breastfeeding. This highlights the need to first train health professionals and then transmit nutrition-related knowledge at the community level. We recommend that further studies could investigate the variables that may impact the nutritional quality of infant feeding practices in the Namuno district. Future research should also assess the impact of myths and taboos. Ultimately, policies aimed at enhancing the local health system and social protection are needed to promote healthy IYCF practices.

## Data Availability

The data that support the findings of this study are available from the authors upon reasonable request.

## Abbreviations

CF: Complementary feeding
CI: Confidence interval
EBF: Exclusive breastfeeding
IYCF: Infant and Young Child Feeding
LMICs: Low-and middle-income countries
MAD: Minimum acceptable diet
MDD: Minimum dietary diversity
MMF: Minimum meal frequency
OR: Odds ratio
SD: Standard deviation
UNICEF: United Nations Children’s Fund
WHO: World Health Organization
